# Editorial: FNIRS in neuroscience and its emerging applications

**DOI:** 10.3389/fnins.2022.960591

**Published:** 2022-08-03

**Authors:** Ning Liu, Meryem Ayşe Yücel, Yunjie Tong, Yasuyo Minagawa, Fenghua Tian, Xianchun Li

**Affiliations:** ^1^Department of Psychiatry and Behavioral Sciences, Stanford University, Stanford, CA, United States; ^2^Neurophotonics Center, Biomedical Engineering, Boston University, Boston, MA, United States; ^3^Weldon School of Biomedical Engineering, Purdue University, West Lafayette, IN, United States; ^4^Department of Psychology, Faculty of Letters, Keio University, Tokyo, Japan; ^5^Department of Bioengineering, University of Texas at Arlington, Arlington, TX, United States; ^6^Shanghai Key Laboratory of Mental Health and Psychological Crisis Intervention, Affiliated Mental Health Center (ECNU), School of Psychology and Cognitive Science, East China Normal University, Shanghai, China; ^7^Shanghai Changning Mental Health Center, Shanghai, China; ^8^Institute of Wisdom in China, East China Normal University, Shanghai, China

**Keywords:** functional near-infrared spectroscopy (fNIRS), methodology, Brain Computer Interfaces (BCI), hyperscanning, multi-modal imaging, frequency-domain fNIRS, resting-state connectivity

This Research Topic focused on recent developments in methodologies and applications of functional near-infrared spectroscopy (fNIRS). It consists of 28 articles on various aspects that were contributed by more than 150 authors. It includes original research articles (24), clinical trials (1), hypothesis and theory (1), and reviews (2).

Near-infrared spectroscopy has been used to study the function of the brain for more than three decades. In recent years, significant progress has been made in methodologies and applications of this technique because of its competitive advantages, such as non-invasiveness, cost-effectiveness, and portability. In this Research Topic, we saw a wide range of novel fNIRS applications among which are the neural mechanism of fantasy (Li et al.), Tai chi chuan (Yang et al.), Haptics-assisted mediation (Zheng et al.), sensory conflict (Nguyen et al.), mental rotation (Mutlu et al.), fatiguing handgrip (Urquhart et al.), and dumbbell exercise (Wang et al.). For instance, Yang et al. reported that Tai chi chuan intervention for 8 weeks could improve ability of inhibitory control in older adults, which was associated with increased prefrontal activation. The findings suggest that Tai chi chuan exercise could be an effective, suitable intervention for improving executive function in elderly people. Zheng et al. reported that haptics-assisted mediation could reduce mind-wandering and enhance attention after a 5-day practice. Moreover, such improvement was related to enhanced activity in the right prefrontal activation and significant changes in the functional connectivity between the brain regions related to the attention networks. The study by Urquhart et al. investigated the effect of motor task fatigue on brain hemodynamics at different frequency bands (endothelial, neurogenic, and myogenic). One strength of this study is that four different types of functional connectivity metrics were applied to the fNIRS signals. Their methods were feasible and revealed that physical activity has an effect on the cortical networks at different frequency bands. Although this study involved healthy adults, these methods were revealed to be applied to older populations and those with impaired cardiovascular health.

The technique was also used to classify second-language proficiency (Lei et al.), monitor virtual reality (VR) based training (Aksoy et al.), monitor transcranial infrared laser stimulation (TILS) (Holmes et al.), and improve acoustic therapy (Sun et al.). Additionally, multiple fNIRS applications on atypical populations have also emerged, including attention deficit hyperactivity disorder (ADHD) (Sutoko et al.), children with Down's syndrome (Xu et al.) and autism spectrum disorder (ASD) (Xu et al.). In the study by Xu et al. fNIRS was used to probe the olfactory function in teenagers with ASD. Measurements of the olfactory function, including odor sensitivity, were conducted by using a fragrance pulse ejection system that not only provides precise amount of odor stimuli, but also motivates and helps individuals with ASD to complete the odor perception task. The use of this system that is exclusively compatible with fNIRS made functional measurement of olfaction possible in individuals with ASD and yielded valuable results. They found significantly weaker activation in the right dorsolateral prefrontal cortex (rDLPFC) in the ASD group than in the neuro-typical group, and the strength of the rDLPFC activity significantly correlated with odor sensitivity. These findings indicate possible impairments in the higher order functions of olfaction, such as olfactory working memory, in individuals with ASD.

FNIRS-based Brain Computer Interfaces (BCI) have gained significant momentum in recent decades. Some notable new developments in this field are the GLM improvement in BCI (von Lühmann et al.), time-resolved fNIRS for BCI (Abdalmalak et al.), and ensemble classifiers for fNIRS-based BCI (Shin and Im). In addition, Nagels-Coune et al. tried to help people with “locked-in” syndrome to communicate with the help of fNIRS-based binary communication paradigm. They have successfully tested the paradigm on healthy participants performing two mental imagery tasks. The study shows the potential of answer encoding using spatiotemporal fNIRS signal features or spatial fNIRS signal features only. Also, Benitez-Andonegui et al. examined an augmented-reality (AR) fNIRS-based BCI on healthy participants that performing motor-imagery tasks. They demonstrated for the first time that by using AR feedback and flexible choice encoding in form of search trees, they can increase the degrees of freedom of a BCI system.

FNIRS-based hyperscanning shows interbrain neural synchrony during social interaction. The field has increased dramatically in the recent decade (Gvirts and Perlmutter, 2019; Balters et al., 2020). Because of its unique data analysis algorithm (Cui et al., 2012), it allows researchers to design tasks with greater flexibility and in more naturalistic settings than typical single-person cognitive studies. In this topic, Dravida et al. investigated joint attention hyperscanning, and they found that social joint attention task induced greater activity in right temporoparietal junction than the non-social condition. Meanwhile, eye-contact frequency could modulate the joint attention activity. More interestingly, cross-brain coherence analysis revealed greater coherence between high eye contact dyads than low eye contact dyads. Cheng et al. studied how the interpersonal coordination pattern modulates coordination outcome and the related brain-to-brain connectivity in dyads that performed a co-drew task. They found that interpersonal multifrequency coordination pattern facilitates the coordination efficiency, which was associated with the enhanced brain-to-brain connectivity. Li et al. reported hyperscanning study of basketball players, and they found that significant interpersonal neural synchronization (INS) was observed in the dorsolateral prefrontal area only in dyads of basketball players when they performed joint-drawing task, which provided the inter-brain evidence for enhanced cooperative behavior in the individuals with team-based sports training.

Multi-modal brain imaging can supplement drawbacks of using a single modality by extracting complementary features. In this topic, Lee et al. examined how the sleeping state influences the resting-state networks (RSNs) in the neonate population using fNIRS-EEG combined method. Neuroimaging for neonates is usually performed when they are asleep; however, RSNs depending on the sleeping state, i.e., quiet sleep (QS) and active sleep (AS), remain unknown. The authors found sleeping state-dependent RSNs with strong long-range connectivity during AS and enhanced short-range connectivity during QS. This finding can significantly contribute to future neuroimaging studies in neonates. Aihara et al. applied their recently developed Hierarchical Bayesian (HB) algorithm on resting state data collected by fNIRS. They evaluated and compared the reconstruction performances of their algorithm with two current algorithms for diffuse optical tomography using fMRI signals as a reference. They showed that their HB algorithm outperformed the prevailing algorithms with higher similarity to the resting state connectivity results from fMRI. Furthermore, Zhang and Zhu reported multimodal fNIRS-EEG recording to study dynamic resting-state connectivity.

In methodological development, Novi et al. revisited the reproducibility of the fNIRS signal during a finger-tapping task, and described a method to increase reproducibility in fNIRS by applying an MRI-based neuronavigation approach. As an emerging neuroimaging technique, the reproducibility of fNIRS signal is still a subject of debate. Earlier studies suggest that fNIRS signal is reproducible over group analysis, but the inter-subject and within-subject reproducibility remains low. Novi et al. revisited this subject and presented a real-time neuronavigation protocol to guide probe positioning. Based on this protocol, consistent, and robust activation of primary motor cortex was obtained at the intra-subject level. Overall, their findings support that integration of spatial information could increases the reproducibility of fNIRS signal.

Lastly, this special issue includes two review articles. Fantini and Sassaroli reviewed Frequency-Domain fNIRS (FD-NIRS) from the basic principles to some most recent developments. They reviewed the absolute optical properties and hemoglobin concentration values reported in the literature for animal models and for the human brain. They also reviewed the application of FD-NIRS on studying hemodynamic responses to brain activity (slow signals) and on studying neuronal activation (fast signals). Furthermore, the authors reported some recent developments of FD-NIRS to maximize the sensitivity to cortical brain tissue relative to the superficial extracerebral tissue (scalp, skull, etc.). Hu et al. reviewed the recent fNIRS studies in brain connectome and resting-state connectivity, with a focus on developing brain. Early brain development from infancy through childhood is closely related to the development of cognition and behavior in later life. Resting-state functional near-infrared spectroscopy (fNIRS) has shown valuable potential in exploring brain network architecture and its changes during the development. They reviewed recent neural developmental research using resting-state fNIRS, both in typical and atypical development of brain connectome. The remaining challenges and future directions in this field were also discussed.

To summarize, the series of research, clinical and review articles that compose this Research Topic covered a wide range of applications and methodologies in fNIRS technique. We hope that this Research Topic will inspire the scientific community with more potential applications, overcome some shortcomings, and promote the technique into more scenarios that could improve the quality of life that span from neonates to elderly adults for both neuro-typical and atypical populations.

## Author contributions

All authors listed have made a substantial, direct, and intellectual contribution to the work and approved it for publication.

## Conflict of interest

The authors declare that the research was conducted in the absence of any commercial or financial relationships that could be construed as a potential conflict of interest.

## Publisher's note

All claims expressed in this article are solely those of the authors and do not necessarily represent those of their affiliated organizations, or those of the publisher, the editors and the reviewers. Any product that may be evaluated in this article, or claim that may be made by its manufacturer, is not guaranteed or endorsed by the publisher.
